# Exploring frame conflicts in the development of a new mineral resource policy in Austria using Q-methodology

**DOI:** 10.1007/s13280-022-01761-9

**Published:** 2022-09-17

**Authors:** Marie-Theres Kügerl, Andreas Endl, Michael Tost, Gloria Ammerer, Philipp Hartlieb, Katharina Gugerell

**Affiliations:** 1 Chair of Mining Engineering and Mineral Economics, Montanuniversität Leoben, Franz Josef-Straße 18, 8700 Leoben, Austria; 2 Resources Innovation Center, Montanuniversität Leoben, Franz Josef-Straße 18, 8700 Leoben, Austria; 3 Department of Landscape, Spatial- and Infrastructure Sciences, Institute of Landscape Planning, University of Natural Resources and Life Sciences Vienna, Gregor Mendel Straße 33, 1180 Vienna, Austria; 4 Department of Socioeconomics, Institute for Managing Sustainability, Vienna University of Economics and Business, Welthandelsplatz 1, Building D1, 1020 Vienna, Austria

**Keywords:** Frame conflicts, Land-use planning, Mineral resources, Mineral resource governance, Mineral resource policy, Q-methodology

## Abstract

Mineral resource policy deals not only with industrial and trade aspects but is nowadays also increasingly concerned with topics such as environmental protection and social development. The tensions associated with strongly divergent opinions among various stakeholder groups cast it as a ‘wicked problem’. The process of redeveloping the ‘Austrian Mineral Resources Strategy’ allows for examination of the different frames and identification of potential frame conflicts in consultation processes at the national level. Using Q-methodology, this paper reveals four frames represented in the Austrian policy design process and unravels conflicts between economy-focused and integrated policy framings. The predominance of the first frame indicates a narrow sectoral approach favouring domestic mineral extraction and economic growth over other means (e.g. international distributional conflicts of benefits and burdens). The article also demonstrates the suitability of Q-methodology as a tool to investigate frames and to improve their understanding in policy design processes.

**Supplementary Information:**

The online version contains supplementary material available at 10.1007/s13280-022-01761-9.

## Introduction

Multiple extant and impending crises related to the long-term functioning of the Earth’s bio-geophysical systems which support human society demand solutions that effectively address natural resource governance (Xu et al. 2020; Rockström et al. 2021). Despite the urgency of this imperative, both global and national arrangements struggle with this wicked governance problem (Rittel and Webber 1973) characterised by key dynamics such as political and organisational inability (e.g. absence of coherent rules and institutions sustainably governing the transboundary flow of minerals), and poly-rationality of stakeholders (i.e. balancing the short- and long-term interests of local communities, national governments, and multi-national mining and original equipment manufacturer (OEM) corporations). Difficulties are expressed through rising societal concerns and civil society protests criticising the dominance of economic interests in the policy discourse at the expense of concerns around environmental protection, ecological integrity, or human rights (Bolger et al. 2021). Accounting for and balancing these key dynamics requires suitable natural resource management approaches.

Against this background, challenges for global resource governance involve issues of limited involvement, connectivity, and collaboration of stakeholders with different backgrounds, values, and mental frames in policy- and decision-making processes, including the (mis)alignment of visions and objectives across institutions (Potts 2020). The constellation of divergent stakeholder perspectives, problem understandings, and values, as well as scientific uncertainty and the need for transformational knowledge, define the challenge of mineral resource governance as a wicked problem (Endl 2017). This challenge is exacerbated by the fact that issues defining resource governance are framed through a variety of communication channels, knowledge structures, paradigms, or stakeholder views (e.g. Dewulf et al. 2009; Davies et al. 2016). Overall, this makes agenda setting and designing, and agreeing on policy interventions a particularly challenging if not irreconcilable task.

Increasingly globally connected supply chains and growing recognition of their potentially negative impacts on the environment and societies, coupled with distrust and the ineffectiveness of private sector responses, has led to the emergence of new governance arrangements on national (McAllister et al. 2014; Endl et al. 2018) and international scales (Ali et al. 2017; Henckens et al. 2019). These are characterised by increasing integration of different policy resorts, multi-stakeholder perspectives, consideration of environmental limits, and new visions for societal and sustainable development. Accordingly, various policy initiatives to improve resource governance have emerged in the European Union and its Member States (MS) over the last two decades. The topics covered include domestic mineral extraction, secondary raw materials, and circular economy (CE) (e.g. the European Raw Materials Initiative (RMI) or the European Raw Materials Alliance (ERMA)), as well as due diligence and responsible sourcing in international mineral value chains that directly or indirectly influence nation states in Europe and beyond (e.g. the EU Conflict Minerals Regulation). On EU policy level, the RMI has played a pivotal role in mineral resource policy making by focusing on activities around the three pillars of (i) domestic mineral extraction, (ii) access to international sources, and (iii) resource efficiency, recycling, and decreased consumption (EC 2008; Christmann 2021). This strategy is complemented by the European Innovation Partnership on Raw Materials (EIP-RM 2013) and its Strategic Implementation Plan (EIP-RM SIP) consisting of 95 specific implementation actions across seven priority areas, including supply security (e.g. improving supply conditions and diversification of raw materials sourcing), resource efficiency, substitution, and environmental, social, and health impacts (EIP-RM 2013; Christmann 2021).

Austria’s first Minerals Strategy (AMS 2010) and Mineral Resources Plan (AMRP 2010) were directly linked to the RMI and transposed its three main pillars into national policies. During the last decade, the AMRP and its policy design process have been celebrated as a European best-practice exemplar showcasing the successful integration of different policy interests and stakeholder involvement (Weber et al. 2008; Tiess 2010; Stiftner 2014). Hence, Austria has undertaken a vivid policy development process over the last twenty years and, thus, has been subject to many different discourses pursuing their integration into a coherent policy approach. Nevertheless, the body of research on Austria’s mineral resource policies, particularly the policy design processes, remains limited and represents an important research gap. The consultation process for the ‘Austrian Mineral Resources Strategy 2030’ (AMRS 2030) was initiated in 2020. Our participation in an international research project gave us access to this ‘by-invitation-only’ policy development process in order to investigate framing issues during the agenda setting stages.

Our research aims to generate knowledge on policy framing among diverse stakeholders and frame conflicts occurring in the agenda setting phase. The article starts by outlining the academic discourse in which our work is embedded, followed by a summary of the EU and national policy frameworks. We then explain the policy development process for the AMRS 2030 and describe the methodology with which we address hypotheses derived from state-of-the-art resource governance models and contemporary discourses on mineral-specific approaches on international, EU, and national levels. The paper closes by discussing the implications of the results, which show that the AMRS 2030 takes a rather narrow sectoral approach, leading to the underrepresentation of distributional conflicts concerning the benefits and burdens of resource extraction and consumption.

## Theoretical framework

While the current academic discourse highlights underlying principles, requirements, or characteristics of effective resource governance (Lockwood et al. 2010; Potts 2020), less emphasis is placed on analytical and normative dimensions of political processes in framing resource governance approaches (Pahl-Wostl 2007). Furthermore, mineral resource governance has received much less attention than resource categories such as water, biodiversity, or forestry (e.g. Hammer et al. 2011; Schultz et al. 2021). Only few studies investigate mineral resource governance on the global or supra-regional level (Küblböck 2015; Ali et al. 2017; Henckens et al. 2019; Ayuk et al. 2020; Christmann 2021), while even fewer investigate mineral resource governance and its implementation on national and sub-national levels (Haikola and Anshelm 2016; Endl 2017; Hidayat and Pramadi 2019).

Some scholars posit that “politics of subterranean materialities” (Marston and Himley 2021, p. 1) or “political ecologies of the subsoil” (Bebbington et al. 2013, p. 274) are forms of human and nonhuman relations to the ‘underground’ (e.g. mineral extraction). These subterranean relations can lead to socio-economic transformations (e.g. mining regions with new forms of social organisation, procurement, and socio-economic development), reiterating the importance of material flows across vertical and horizontal frontiers and boundaries. Considering these subterranean political realities in the context of global supply chains and the current sustainable development agenda has led to increasing calls for international mineral resource governance approaches (Giurco et al. 2014; Ali et al. 2017; Henckens et al. 2019).

The discourse on mineral resource governance originates in the regress of the public sector from managing extractive sectors at the beginning of the twentieth century, leading to more corporate-centred governance (Himley 2010). Subsequent decades have been characterised by various levels of community and societal distrust of the extractive sector (Conde 2017) met with ineffective industry responses (Kemp and Owen 2018), as well as non-existent or weak state governance in some countries (Himley 2010). The high level of complexity in managing societal expectations regarding mineral extraction has led to the emergence of new governance arrangements on national (McAllister et al. 2014; Endl et al. 2018) and global scales (Ali et al. 2017; Henckens et al. 2019).

Academic discourses also reflect immanent tensions and provide a critical view on (i) distributional conflicts regarding benefits and burdens arising from resource extraction and use in different parts of the world, including discourses around the ‘resource curse’ or green/resource colonialism (Broad and Fischer-Mackey 2017; Hilson and Maconachie 2020), and the ‘imperial mode of (production and) living’ of the ‘western’ industrialised world (Scheidel et al. 2018; Brand et al. 2021); (ii) greening the economic system via modernisation, green capitalism, or green economic development to achieve a ‘sustainable society’ (United Nations Environment Programme and International Resource Panel 2011; Lorek and Spangenberg 2014; Smith 2016); (iii) ecological justice, such as the argument that, legally, mining should not be carried out when causing serious harm to humans and other beings (Sbert 2020); (iv) different governance approaches addressing these issues, varying from international frameworks (Vasseur et al. 2017; Ayuk et al. 2020) to national approaches on trade politics (Küblböck 2015; Barteková and Kemp 2016) or sustainability in mineral resource policy (Haikola and Anshelm 2016; Mancini and Nuss 2020; Janikowska and Kulczycka 2021).

### The current discourse on mineral resource governance in the European Union and globally

Mineral resource governance has emerged as a contested topic over the last two decades, both at the EU level and globally. The concept has recently been presented by the UN International Resource Panel on the international political agenda (Christmann 2021) and has its place in EU level and MS policy discourses due to the RMI and ERMA (EC 2008; Schäfer et al. 2020). The EU steer MS mineral policies either (i) directly, using soft tools such as voluntary frameworks (e.g. RMI) which respond to global challenges, including supply security or market distortions (Schäfer et al. 2020), or (ii) indirectly through mandatory instruments such as environmental protection and human health and safety directives and regulations which impact extractive activities (Scannell 2012). While EU governance on domestic extractive activities is restricted to ‘incentivising’ or guiding due to the sovereignty of its MS, it plays a very active and influential role in other policy areas, such as due diligence in global mineral value chains, CE, and secondary raw materials (e.g. battery production standards) (Christmann 2021). Hence, mineral resource governance is strongly shaped by the EU policy agenda beyond domestic mineral extraction at the MS level (Smol et al. 2020).

Given the transboundary nature of global value chains (Henckens et al. 2019), mounting concerns over supply security (Barteková and Kemp 2016), and increasing demands for due diligence considering upstream actors in global mineral supply chains, various actors are moving towards more integrated mineral resource governance approaches. Nation states or supra-regional bodies such as the EU strategically position mineral resource extraction next to concerns regarding security of supply, competitiveness, resource circularity and recycling, and other sustainability issues along the entire value chain (Schäfer et al. 2020; Christmann 2021). This discourse has recently focussed on responsible sourcing, which emphasises the responsibility of downstream players and other parts (e.g. service providers and traders) of mineral value chains.

Recent academic discourses have conceptualised various ‘integrated’ governance regimes for mineral resources (Nickless 2017; Ayuk et al. 2020), including an expanded Social Licence to Operate (SLO) concept (‘Sustainable Development Licence to Operate’—SDLO), which acknowledges the global character of environmental issues through “unequivocal recognition of planetary boundaries” (Ayuk et al. 2020, p. 12) and implies greater recognition of sustainable development goals and societal concerns in the extractive sector. Other governance regimes attempt quadruple bottom-line accounting by considering “economic outcomes, sound environmental management, the respect of social values and aspirations, and adherence to the highest standards of governance and transparency” (Ayuk et al. 2020, p. 8), yet it remains unclear how distributional conflicts are framed in these more integrated mineral resource governance approaches, and how the framing affects the policies ultimately enacted in nation states.

### The framing of issues and conflicts in agenda setting for mineral resource governance

Against this background, our research examines agenda setting in public policy design processes by investigating the existence of different frames and their relation to various aspects of mineral resource governance. Kingdon and Stano (1984, p. 5) define a policy agenda as “the list of subjects or problems to which governmental officials, and people outside of government closely associated with those officials, are paying some serious attention at any given time”. In this regard, the identification of ‘frames’ and insights into their alignment, co-existence, and potential conflicts (van Hulst and Yanow 2016) can inform agenda setting and the design of policies and/or aid in the understanding and interpretation of such outcomes. The cognitive-representational stance on framing, originally discussed by Bateson in 1954, focuses on the “way that people experience, interpret, process or represent issues, relationships and interactions in conflict settings” (Dewulf et al. 2009, p. 160). With regards to public policy, van Hulst and Yanow (2016, p. 105) take the view that policy public administrators “are not always cognizant that problem definitions are not given, but ‘framed’, let alone aware of how such framing takes place”. The consideration of framing is recognised by van Hulst and Yanow as a reflective process which adds value in a policy discourse arena increasingly defined by networked governance approaches to solving complex or wicked problems. In this context, the analysis of frames serves as a useful means to discern how certain issues are perceived or strategically hidden (Cairns and Stirling 2014). Schön et al. (1996) conceptualise effective policy design as a co-design process in which frame conflicts are either ‘pragmatically resolved by reframing’ or ‘frame reflection is central to design rationality’. Thus, making existing frames and possible frame conflicts visible and tangible is relevant for both agenda setting and the later policy design phase, particularly in dealing with ‘wicked’ policy problems involving conflicting paradigms and worldviews that require deliberative and participatory approaches to resolve them (Biermann et al. 2017; Patterson et al. 2017). The arena of mineral resource governance is marked by exactly such controversies, conflicts, and trade-offs across its different dimensions (Nickless 2017; Ayuk et al. 2020). The frames originating from such a process might differ significantly, reflecting divergent economic, social, environmental, and justice priorities, and potentially reveal new tensions (Dewulf et al. 2009; Davies et al. 2016). Wicked problems such as resource governance nevertheless require the involvement of diverse stakeholders to account for their complexity. More specifically, they require a (policy) process that adequately incorporates divergent interests and accounts for their sometimes conflicting nature as well as differences in the agency of various stakeholders to exert influence during both the problem framing and solution phases. Concluding, the consideration of frame conflicts and their implications is highly relevant for agenda setting and policy design in mineral resource governance processes purposefully engaging in inclusive multi-stakeholder approaches (Bulkeley and Mol 2003).

### EU mineral resource goverance and its influence on Austria’s mineral policy

For more than a decade, a pivotal aim of EU foreign policy has been to secure access to mineral resources that are vital for the EU economy (EC 2008; Küblböck 2013). The RMI (2008) is a core EU policy which targets three areas: (i) to provide undisturbed access to international markets, (ii) foster domestic mineral resource extraction by adapting framework and institutional conditions, and (iii) reduce consumption of primary materials through circular economy or design for recycling, plus the identification of critical raw materials as an additional policy objective (EC 2008). This policy has not been updated since 2008 yet is implemented via a range of policy measures (e.g. trade and investment policies, mineral resource policies, land-use planning instruments) on EU and/or MS levels to ensure a common and integrated approach (Küblböck 2013; Gugerell et al. 2020).

Austria implemented its first mineral resource policy in 2010, consisting of two main policy instruments: the AMS and the AMRP (Weber et al. 2012). The main priority of the AMS 2010 was to ensure a sustainable supply of primary and secondary raw materials for Austria’s industry (Weber et al. 2012), echoing the three pillars of the RMI. This was complemented by the AMRP 2010, a policy instrument aiming to identify ‘conflict-free’ domestic deposits and safeguard these mineral deposits and their future access (Holnsteiner 2015). Austria’s competent authority is the ‘Mineral Resources Policy’ Department (D IV/5) of the Federal Ministry of Agriculture, Regions, and Tourism (BMLRT).

Compared to other policy areas such as climate governance (Steurer and Clar 2015; Niedertscheider et al. 2018), the discourse on mineral resource governance is less developed in Austria and poses a research gap. Scholarly work mainly addresses mineral resource policy (Wagner et al. 2006; Tiess 2010), valuation criteria to identify mineral deposits for safeguarding (Weber et al. 2008; Carvalho et al. 2021), and policy integration and implementation (Endl 2017; Endl et al. 2020; Gugerell et al. 2020). While the AMRP 2010 was and still is celebrated as best practice regarding its method of mineral deposit valuation and its strong policy coordination (e.g. with land-use planning, biodiversity conservation, water policy, agriculture), policy delivery has been impaired by a highly fragmented approach to the actual implementation (Rechnungshof 2017; Gugerell et al. 2020).

The EU has launched various policies over the last decade that impact the mineral resource sector, including the European Green Deal with its core goal of net-zero emissions by 2050. The Green Deal is complemented by the Circular Economy Action Plan, the Roadmap to a Resource-Efficient Europe, and a new European Industrial Strategy that should support the transition to a green economy. While this economic transition drives the demand for primary resources, these policies also target the decoupling of economic growth and resource use (Smol et al. 2020). Additionally, an increasing number of policies have been issued to promote responsible sourcing of mineral resources for EU industries (Mancini and Nuss 2020), such as the Conflict Minerals Regulation, the Strategic Battery Action Plan, the upcoming Corporate Sustainability Due Diligence Directive, and the Critical Raw Material Action Plan. The latter also includes the establishment of ERMA focussing on increasing the EU’s resilience in mineral value chains (EC 2020). These changes in the EU policy landscape in combination with the UN Sustainable Development Goals (SDGs) require adequate responses from MS (Smol et al. 2020).

### Austria’s mineral resource strategy 2030

In the context of the new EU policy landscape, the BMLRT proposed a fundamental revision and realignment of the existing mineral resource strategy and started the design process for the AMRS 2030 in spring 2020. The initial phase pursued the development of an ‘Integrated Austrian Mineral Resource Strategy’ (Köstinger 2019), but the 'integrated' aspect has receded into the background over time and disappeared altogether from the current working title: ‘Austrian Mineral Resource Strategy 2030’ (BMLRT 2020). It was expected that this new strategy would again mirror the original three RMI objectives (EC 2008) as well as including additional aspects such as sustainability, circular economy, awareness raising regarding the importance of mineral resources, increased public acceptance of mineral resource extraction, digitalisation, and innovation. This set of characteristics is intended to propel Austria into a pioneering role in European industry through technological development, innovation, and improved international competitiveness (BMLRT 2020). Explicitly, the policy should: (i) contribute to an expansion and increased attractiveness of Austria as a business location; (ii) ensure the sustainable and efficient use of raw materials within the framework of a circular economy and by applying innovative technologies; (iii) strengthen the resilience of the raw material sector and value chains to ensure a responsible and secure supply of primary and secondary raw materials; and (iv) secure prosperity and an increasing quality of life for the Austrian population (BMLRT 2020).

### The consultation process for the AMRS 2030 policy design

To assist the policy design of the AMRS 2030, the BMLRT launched a ‘by-invitation’ consultation process consisting of ten thematic workshops in June and July 2020. Representatives from mineral resource administration, industry, mineral resource related lobbies/chambers, and from selected academic and research organisation were invited (see Method and Data Collection and Fig. 2 for the distribution). Civil society organisations (CSOs) or representatives from neighbouring policy fields such as environment and climate were not invited.

Over six workshops, the participants worked on topics that resemble the three RMI pillars: (i) sustained supply from domestic sources and (ii) international sources, and (iii) smart production. These topics were complemented by four cross-cutting workshops addressing: (i) digitalisation and automation; (ii) science, innovation, and technology; (iii) education, stakeholder dialogue, and raising awareness for mineral resources; and (iv) foresight policy and EU and international mineral resource policy. Ahead of the workshops, input papers were circulated that provided a comprehensive overview of the workshop theme, tensions, possible solution spaces and measures, as well as guiding questions for the workshop. Each workshop was facilitated by a moderator and a rapporteur who documented the results and informed the involved ministerial department on the output, while there were no feedback loops towards the participants. The output documents illustrate the governance topics advocated by participants: (i) sustainability in mineral extraction and smart production, (ii) decarbonisation at an economically justifiable level, (iii) securing long-term accessibility of deposits, and (iv) relegation of compensatory measures and landscape levies such as taxation of mineral raw materials, which are considered competitive distortion (BMLRT 2020).

## Materials and Methods

Q-methodology is a well-established, explorative, semi-quantitative methodological framework to canvas a certain population’s distinctive frames “of subjectivity across an increasing array of human activities, most recently including decision making” (Brown 2019, p. 1). Q-methodology has been employed across a variety of research fields, including resource conservation (Benitez-Capistros et al. 2016; Lauret et al. 2020), resource management and mining (e.g. Davies et al. 2016; Nguyen et al. 2018), and public policy studies (e.g. Brown, 2019). A core component of the Q-methodological procedure is the Q-set, which is a collection of objects —often statements— that comprehensively address the topic under investigation (Sæbjørnsen et al. 2016; Braito et al. 2020). Rigour and care are important for the construction of the Q-set, since it plays a central role in the data collection and, thus, has substantial impact on the quality of results (Baker et al. 2017). This research closely follows the established approach by Watts and Stenner (2012) and Brown (2019).

### Q-sampling and Q-set development

The initial step in developing a Q-set is to establish the concourse: a set of statements that comprehensively represents the sum of the discourse on the given research topic. The concourse for this Q-set is constructed from the input and output documents from the consultation process, complemented by extracts from pertinent civil society debates in Austria and the EU (AG Rohstoffe 2019; European Environmental Bureau (EEB) (ed) 2020). The concourse was structured into ten thematic fields following the topics of the consultation process workshops and statements were added to all fields to develop a first Q-set (Watts and Stenner 2012).

The initial Q-set was developed following a naturalistic Q-sample approach (Sæbjørnsen et al. 2016) with 146 statements (Table 1). In five internal review rounds, the research team refined, condensed, and reduced the statements to a manageable number of 40–80 (Watts and Stenner 2012). The team additionally validated the Q-set with external experts from public administration (BMLRT), who provided feedback regarding clarity, wording, and policy specifics and pre-tested the Q-set. The heterogeneity of the research team, including mining (3 researchers), technical environmental protection (1), policy research (1), and land-use planning (1), safeguarded a balanced reduction and representation of different topics. The final Q-set consists of 54 statements (full list of statements in Appendix S1).

**Table 1:** Thematic fields of initial & final Q-set; initial number of statements per category and elimination process (R1—final Q-set; ministry indicates feedback from public administration experts (BMLRT))

Thematic categories	Initial Q-set	R1	R2	R3 (Ministry)	R4	Final Q-set
Digitalisation	13	10	11	4	3	3
Recycling/Circular Economy	34	15	10	10	7	7
Raw material supply from domestic sources	21	12	3	3	3	3
Responsible sourcing	13	5	6	11	7	7
Value chain	17	6	4	5	5	5
Sustainable supply	10	5	4	3	2	2
Land-use planning	9	3	5	5	5	6
Ecology/Environment	13	9	8	6	6	7
Social Licence to operate	16	13	12	7	6	7
Miscellaneous (e.g. Natural Resource Justice)					3	7
Total	146	78	63	54	47	54

### Data collection—Q-sort technique

Q-methodology does not require a minimum or maximum number of participants since it is not aiming for generalisation or to represent proportions in a population, but rather to establish the existence of particular frames and to characterise these (Watts and Stenner 2012). All individuals that participated in the consultation process (P initial P_i_: *n* = 66, 13 female, 53 male) were invited, of which 41 responded and agreed to participate in the Q-sort (P-set, 60.6% of P_i_): 3 participants from policy/public administration (P_i_: 4), 12 from research and academia (13), 10 from lobby/chambers (21), and 16 from industry (28). One participant dropped out during the Q-sort, resulting in a final P-set of 40.

**Fig. 1:**
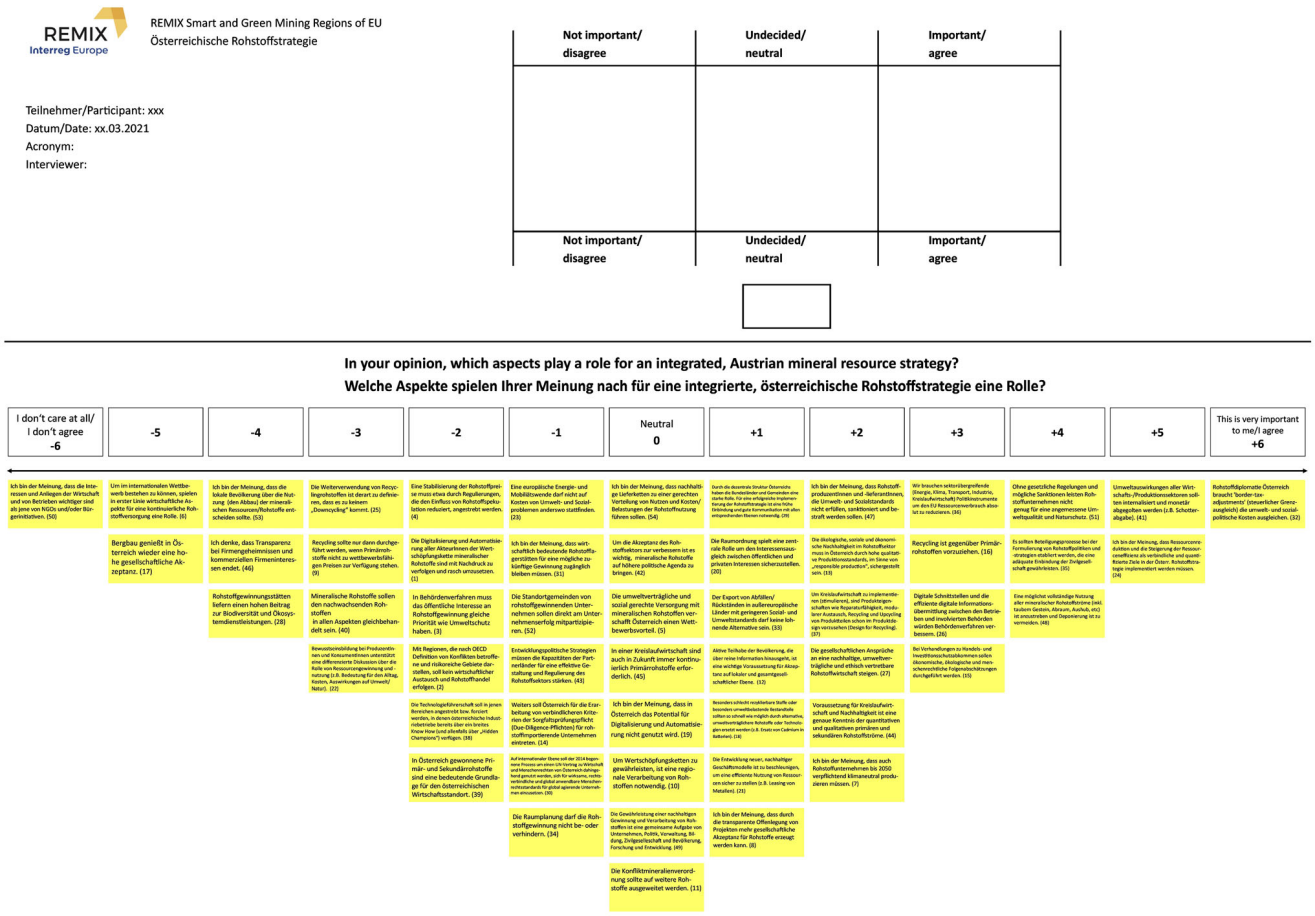
Virtual whiteboard template for online Q-sorts; Top: three piles – not important/disagree, neutral/undecided, important/agree; Bottom: forced-choice distribution from -6 most unimportant/completely disagree to + 6 most important/completely agree; *Note*: The sort on display is for demonstration purposes only and reflects neither the opinion of any participant, nor the authors

The data collection took place between January and April 2021. Due to the Covid-19 restrictions in Austria at the time of the study, the data collection and Q-sorting exercises were conducted online. The Q-set and the sorting template were converted to a virtual whiteboard tool (Fig. 1). We preferred this ‘hands-on’ approach over a digital Q-sort tool, as the interaction with the participants and the ex-post interviews in which participants explain their Q-sort were considered crucial for the interpretation of results. The Q-sorting followed a standardised three-step procedure: (i) introduction and professional or private relation to the topic; (ii) explanation of the method, introduction to the virtual whiteboard tool, and sorting the digital cards into three initial piles (not important/disagree, neutral/undecided, important/agree) (Stenner et al. 2008); and (iii) sorting the three piles into the forced-choice distribution ranging from − 6 most unimportant/completely disagree to + 6 most important/completely agree based on the question “In your opinion, which aspects play a role for an integrated Austrian Mineral Resource Strategy?”, then reviewing the finalised sort to ensure the final configuration accurately depicts their viewpoint. The sorting procedure was complemented by a semi-structured ex-post interview covering an explanation of items placed at the two extremes, identification of possible omissions from the Q-set, and topics the participants consider crucial for an integrated raw materials strategy. Ethical research standards based on the universities’ ethical and legal guidelines, such as informed consent and GDPR regulations, were implemented at the start of each data collection session. The online sessions were recorded, transcribed, and analysed as supplementary material (Kuckartz 2016).

### Q-methodological factor analysis

The 40 Q-sorts from the virtual whiteboard templates were transferred to PQMethod, a free software for the statistical analysis of Q-studies (Schmolck 2021). The Q-sorts were analysed using Principle Component Analysis (PCA) followed by a Varimax Rotation to identify patterns and distinct factors (shared frames). Based on the following selection criteria, factors one, two, three, and five were extracted: (i) Eigenvalue > 1.0, and (ii) at least two Q-sorts loaded significantly on the respective factor. The significance level is calculated using the equation: 2.58 × (1/√No. of items in the Q-set, at *p* < 0.01 level) (Watts and Stenner 2012), resulting in a significance threshold of 0.35 for our study. Following Braito et al. (2020), we raised the significance level to 0.50 to assure a higher resemblance between the Q-sorts loading to the respective factor and, thus, enable each factor (frame) to be more accurately described. The four extracted factors account for 54% (minimum level 35–40%, Watts and Stenner 2012) of the explained variance and 35 defining variables (Table 2). Further analysis was supported by crib sheets (Watts and Stenner 2012) illustrating the highest and lowest ranked statements of each factor (factor arrays), the Z scores (i.e. normalised factor scores), and key aspects from the ex-post interviews. Following Davies et al. (2016), the extracted factors express frames and are approximated by the results of the factor arrays and Z scores.

**Table 2:**
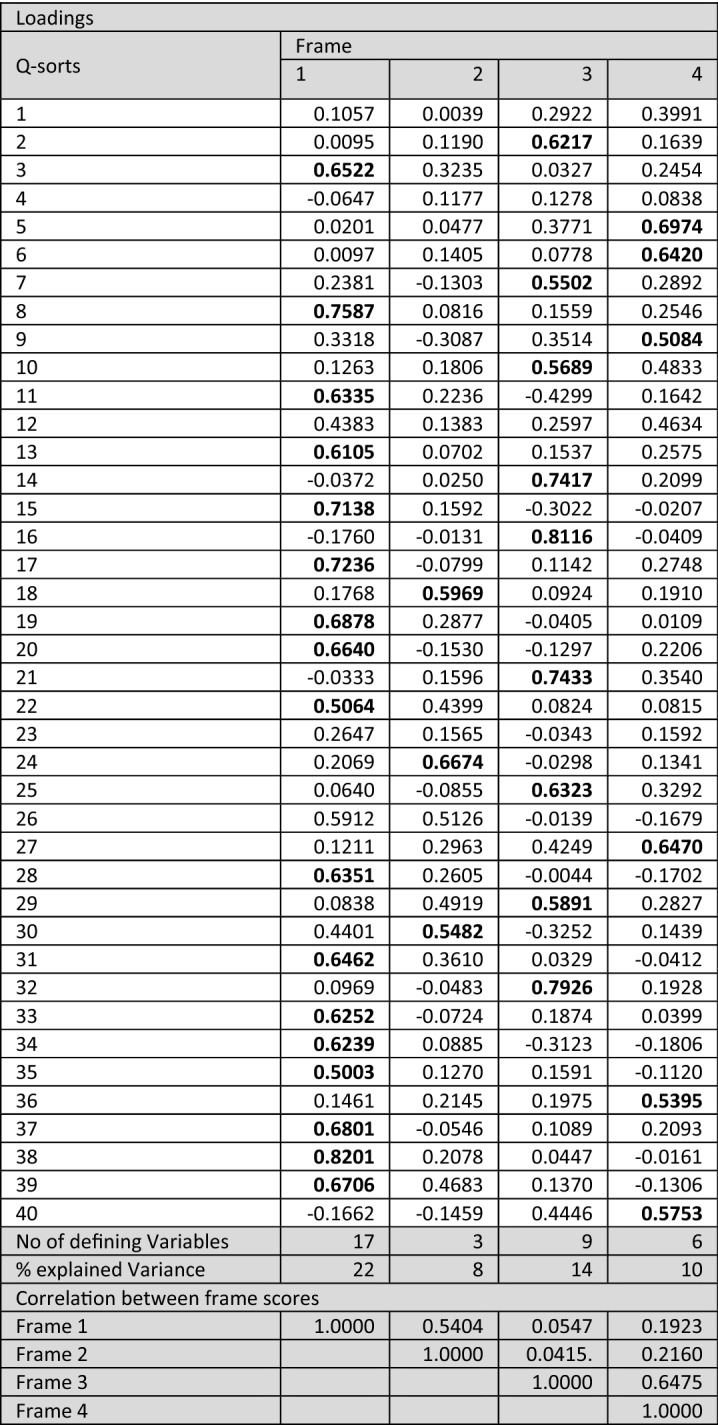

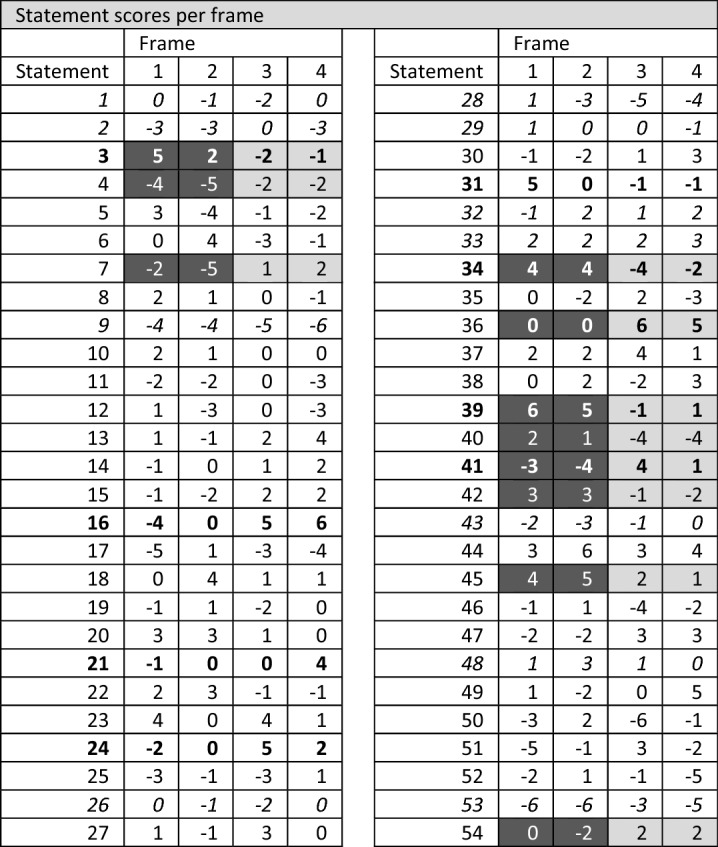
Top: Extracted frames and respective loadings of the 40 Q-sorts (defining factors in bold); Bottom: Statement scores per frame (Factor Arrays) (distinguishing statements in bold, consensus statements in italics, similarities of related frames highlighted in grey)

## Results

**Fig. 2:**
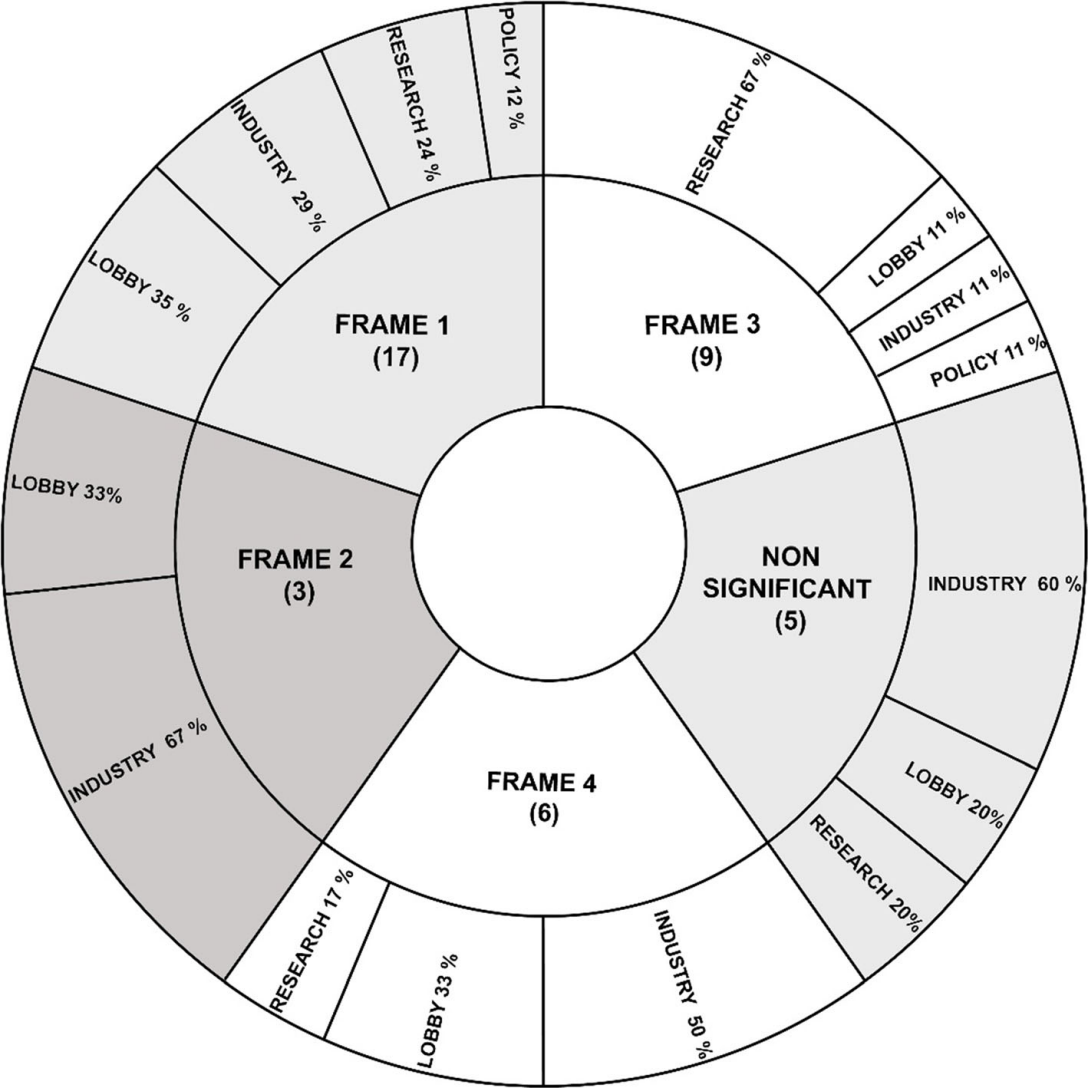
Stakeholder group distribution between frames

The Q-sorting of the 54 Q-statements by 40 participants resulted in four distinct frames (significance level raised to 0.5), with all but five participants loading significantly on one of the four frames. Four participants did not load on any of the frames and one participant loaded on two frames (frames one and two)—they are summarised in the category ‘non-significant’ (Fig. 2). Figure 2 illustrates that representatives of industry and lobbies/chambers are heavily represented in frames one and two, while frame three is populated more by representatives from research and academia and policy/public administration. Frame four includes members of industry, lobbies/chambers, and research and academia, but does not include representatives of the policy/public administration sector. The results show that researchers with a background in traditional mining relate to frame one, while researchers more related to sustainability issues gather in frame three. Similarly, industry and lobby/chamber representatives from the extractive sector mainly load on frame one, while representatives from other industry sectors relate more to frame four.

### Frame one – Continuous economic growth and domestic mineral extraction

Frame one is the most popular viewpoint with 17 defining Q-sorts (Table 2), mainly representing participants from industry and lobbies/chambers as well as researchers with traditional mining backgrounds. It is characterised by a desire for continuous economic growth and a focus on domestic mineral extraction. This frame considers the Austrian raw materials’ industry as the decisive basis for economic development in Austria (statement No 39/rank + 6) that must be promoted by land-use policies (3, 31/+ 5) and public involvement (12/+ 1). The neoliberal thought (Mirowski and Plehwe 2009) of this frame is backed up by the unequivocal rejection of environmental protection and internalisation of externalities (e.g. environmental damage) (41/− 3), instead considering that mining companies are already implementing environmental protection measures and that further regulation is not required (51/-5). The rejection of further regulation is also reflected in the post-sorting interviews with adherents to frame one, who criticised the majority of statements involving regulatory measures. The main environmental consideration in this frame concerns the avoidance of impacts related to the European energy transition somewhere outside the EU (23/+ 4). Compared with the other frames, mandatory implementation of resource reduction targets ranks notably lower (24/− 2), while guaranteeing domestic extraction and processing (10/+ 2) to improve the competitive advantage (5/+ 3) against other economies ranks notably higher. Extractive industries are considered at a disadvantage compared to environmental protection (3/+ 5), which should be changed in the future. Land-use planning is considered a helpful tool to provide access and facilitate mineral extraction, and increase public acceptance (31/+ 5, 34/+ 4, 42/+ 3). The importance of land-use policies for this frame is echoed in the post-sorting interviews, where participants expressed concerns that the strategy could fail if land-use planning does not recognise the importance of mineral extraction and secure access to resources. Digitalisation and responsible sourcing are not important in this frame.

### Frame two – Economic growth and increased role of recycling

Frames one and two are strongly related (correlation of *r* = 0.54: Table 2) and frame two is populated by industry and lobby/chamber representatives. Both frames share the pivotal focus on economic growth, the role of raw material production, the Austrian raw materials industry (39/+ 5) as key to economic prosperity, and the disregard of digitalisation. However, they differ in their assessment of commodity types; recycling and secondary raw materials are more important for frame two in terms of substituting polluting and non-recyclable materials (18/+ 4) and knowledge of material flows (44/+ 6), although increasing recycling activities in general is not considered. The interests of extractive industry and economic actors are prioritised over CSOs’ (50/ + 2) or advancing sustainability (5/− 4, 13/− 1, 27/− 1, 49/− 2), with sustainability, supply chains, and due diligence considered insignificant aspects for Austrian mineral resource policy (15/− 2, 30/− 2, 54/− 2). Environmental and nature protection and related regulatory measures and sanctions (41/− 4), climate neutrality (7/− 5) or supporting developing countries (43/-3) are considered even less important in this frame than in frame one. Responsible sourcing is accepted as long as it represents an economic advantage in international markets (6/+ 4). Representatives of this frame appraise public involvement in decision making (12/− 3) lower than any other frame and clearly reject the concept of 'Free Prior and Informed Consent' (FPIC) in the extractive industries (53/− 6).

### Frame three– Integrated policy for the environment and climate

Frame three is the second most popular with nine defining Q-sorts, mainly representing the viewpoints of participants from research and academia. Different aspects of policy integration play a pivotal role in this frame, including the importance of cross-sectoral policies (36/+ 6), regulatory measures such as the implementation of specific reduction targets (24/+ 5), and sectoral policies on resource efficiency, recycling, and decreased material consumption. The near-zero statistical correlation between frames three and one (*r* = 0.05: Table 2) and between three and two (*r* = 0.04) shows the conceptual contrast between frame three and those previously discussed. While frames one and two focus on the support and growth of the Austrian raw materials industry, ecological and environmental considerations are a main theme of frame three, including the prevention of environmental and social impacts of energy transitions elsewhere (23/+ 4) and the role of policy and policy tools to improve environmental protection and environmental quality (51/+ 3, 41/+ 4). The emphasis on recycling and circular economy and their implementation through policy (instruments) is an essential difference to the first and second frames. Supporting this interpretation, responsible sourcing, penalising the infringement of environmental and social standards (47/+ 3), and attaining higher levels of transparency (46/− 4) are supported. Unlike in frame two, the role of civil society and NGOs (50/− 6) and processes that actively include civil society (35/+ 2) are important. Although not strongly supported, FPIC ranks higher for frame three (53/− 3) than any other frame. When asked what could cause the strategy to fail, representatives of this frame emphasised above all a lack of cross-sectoral involvement, but also the lack of stakeholder participation. Several participants shared the perspective that the strategy would be considered a failure "[…] if you develop a mineral resource policy or a mineral resource strategy as a pure trade and economic strategy only within your own political and stakeholder bubble and then, at the end, the NGOs are allowed to comment a little on it […]".

### Frame four—New business models for recycling and lower consumption

Frame four is defined by six Q-sorts from representatives of industry and lobbies/chambers not directly connected to mining. This frame shows a thematic overlap with the third frame (*r* = 0.65: Table 2), particularly regarding the importance of recycling (16/+ 6, 9/− 6) and reduction of resource consumption in the EU (36/+ 5). In contrast to frame three, however, this frame does not include further (policy) considerations on how to enhance recycling activities beyond generally prioritising secondary over primary raw materials. Moreover, ecology and environmental aspects play a less vital role than in frame three. Only in frame four are items from the ‘value chain’ category represented multiple times at the two extremes. While the development of new business models for more efficient resource consumption are encouraged (21/+ 4), the extension of the Conflict Minerals Regulation (2/− 3) and the restriction of trade with high-risk areas (11/− 3) are not supported. The representatives of frame four consider responsible production and high environmental and social standards as important (13/+ 4) concerns for the Austrian mineral resource strategy, while collaboration of all stakeholder groups to achieve a sustainable raw materials sector (49/+ 5) is seen as crucial. Further results reveal a tension in this frame: while the collaboration of all stakeholder groups is deemed important to achieve a sustainable raw materials sector, one might question how that is possible without transparency (8/− 1), meaningful engagement of civil society in policy design processes (35/-3), and instruments such as benefit sharing (52/− 5), all of which rank significantly lower than in the other frames. Similarly, Social Licence to Operate (SLO) is also ranked in the strongly negative spectrum of this frame. However, policy approaches to achieve high social (30/+ 3) and environmental (7/+ 2, 33/+ 3) standards along the supply chain (14/+ 2) rank higher than in the other frames. Divergent to frame three, responsible sourcing is no pivotal consideration in this frame.

### Commonalities and differences across frames

Neither digitalisation nor its improvement (statements 1, 19, 26) have turned out a core element of any of the four frames. Instead, this topic is positioned in a neutral to slightly adverse range. Apart from digitalisation, eight further statements have similar ranks throughout the different frames (Table 3).

All frames agree (+ 2 or + 3) that the export of waste to countries with lower environmental and/or social standards (33) should be prevented. They also share a positive attitude towards recycling, emphasising that recycling should be implemented and furthered regardless of whether primary raw materials are available and can be sourced on the global market at competitive prices (9). All frames question the importance for the Austrian mineral resource strategy of supporting partner countries’ capacities for effective governance mechanisms (43), which is ranked between neutral and slightly negative.

The strongest deviation between the frames opens up in the question of whether recycling should be prioritised over primary raw materials (16): while frame one opposes this proposition (− 4), frames three and four favour recycling and secondary raw materials over primary ones (+ 5 and + 6). Frames one and two emphasise that primary raw materials will continue to play a pivotal role in the circular economy (45/+ 4, + 5), while the other frames take a more impartial position (+ 2 and + 1).

FPIC (53) is rather disregarded in all frames: frames one, two, and four firmly reject the topic (− 6 or − 5), while frame three shows a relatively more positive attitude towards this statement (− 3).

A category that is on the one hand very unifying but at the same time triggers very pronounced opposition is ecology and environment (3, 7, 36, 41). Frames one and two emphasise that raw materials should enjoy the same policy priority like environmental and nature protection and strongly repudiate climate neutrality of extractive industries by 2050 (7). On the other side, frames three and four strongly endorse the internalisation of environmental externalities and advocate cross-sectoral policies to attain decreased resource consumption (36). The attitudinal difference between these two groups is also pronounced regarding regulatory measures and whether sanctions are suitable measures to ensure environmental quality and nature protection (51); while frame one fully rejects institutions and sanctions (− 5), frame three considers them as essential measures to meet environmental and nature quality and policy goals (+ 3). The limited contribution of mineral extraction to biodiversity and ecosystem services is the only ecological issue the four frames can agree on, despite frame one moderately agreeing that extractive industries have a net positive impact on biodiversity.

The post-sorting interviews show a more mixed picture. Both groups (frames one and two, and frames three and four) agree on what an integrated strategy should not be—it should not preserve the status quo but rather contain concrete, implementable targets. Where the two groups differ, however, is on missing aspects and the characteristics that make an integrated strategy successful. While frame one places great importance on education and awareness raising for the raw materials sector, the acceleration and simplification of official procedures, as well as the role of the domestic (extractive) industry; frame three is mainly concerned with the involvement of all stakeholders, as well as the entire value chain, the equality of primary and secondary raw materials, and the triple-bottom line of economy, ecology, and social issues.

Table 3: Statements of consensus, tension (one rank up or down) or conflict (two ranks up or down) across frames, design based on (Morinière and Hamza 2012).

**Table 3:** Statements of consensus, tension (one rank up or down) or conflict (two ranks up or down) across frames, design based on (Morinière and Hamza 2012) (ranking: strong agreement or disagreement: ± 6–± 4; slight agreement or disagreement: ± 3–± 2; neutral: ± 1 – 0)

Statement (statement number)	Frame 1	2	3	4
Digitalisation				
*Tension* (1) Digitalisation must be pursued and quickly implemented (26) Digital interfaces between companies and authorities for improved efficiency	Neutral Neutral	Neutral Neutral	Slight disagreement Slight disagreement	Neutral Neutral
Recycling/Circular Economy				
*Consensus* (9) Recycling only if prices of primaries not competitive (33) Export of waste to lower standard countries must not be viable *Conflict* (16) Recycling preferred over primaries	Strong disagreement Slight agreement Strong disagreement	Strong disagreement Slight agreement Neutral	Strong disagreement Slight agreement Strong agreement	Strong disagreement Slight agreement Strong agreement
Raw Materials from domestic sources				
*Tension (48)* Fullest possible use of all raw material flows *Conflict* (39) Primary and secondary raw materials basis for Austria as business location	Neutral Strong agreement	Slight agreement Strong agreement	Neutral Neutral	Neutral Neutral
Responsible Sourcing				
*Tension* (32) Border-tax adjustments	Neutral	Slight agreement	Neutral	Slight agreement
Value Chain				
*Tension* (2) No economic exchange with conflict affected areas *Conflict* (21) Accelerated development of new business models	Slight disagreement Neutral	Slight disagreement Neutral	Neutral Neutral	Slight disagreement Strong Agreement
Land-use planning				
*Consensus* (29) Early involvement and communication with all government levels *Conflict* (31) Maintain accessibility of deposits for future (34) Spatial planning impedes mineral extraction	Neutral Strong agreement Strong agreement	Neutral Neutral Strong agreement	Neutral Neutral Strong disagreement	Neutral Neutral Neutral
Ecology/Environment				
*Tension* (28) Extractive sites contribute to biodiversity *Conflict* (3) Mineral extraction same priority as environmental protection (7) Climate-neutral production by 2050 (36) Cross-sectoral policies for reduced consumption (41) Internalisation of externalities (51) Regulations and sanction for environmental protection	Neutral Strong agreement Slight disagreement Neutral Slight disagreement Strong disagreement	Slight disagreement Slight agreement Strong disagreement Neutral Strong disagreement Neutral	Strong disagreement Slight disagreement Neutral Strong agreement Strong agreement Slight agreement	Strong disagreement Slight disagreement Slight agreement Strong agreement Slight agreement Slight agreement
Others				
*Tension* (43) Development policies to support countries’ governance mechanisms (53) FPIC *Conflict* (24) Quantified targets for resource reduction and efficiency	Slight disagreement Strong disagreement Slight disagreement	Slight disagreement Strong disagreement Neutral	Neutral Slight disagreement Strong agreement	Neutral Strong disagreement Slight agreement

## Discussion

Several frames emerged across the field of participants which strongly varied in their perspective on policy priorities for the Austrian mineral resource strategy along the topics of value chain, circular economy/recycling, digitalisation, responsible sourcing, land-use, ecology and environment, and SLO and civic engagement. Generally speaking, the frame conflicts can be summarised as follows:

i. Predominance of a strongly market-oriented frame based on neoliberal thought (frames one and two) comprehending mineral resource policy primarily as an industrial and trade policy for accessing mineral resources with less restrictive but more supportive regulatory measures for economic development.
ii. In comparison, a conflictual frame (frames three and four) that focuses on societal dimensions, such as public involvement, environmental protection, procedural justice (SLO) paired with characteristics of lower material consumption and a specific focus on secondary raw materials and recycling; this set of characteristics is largely rejected by the other two frames.
iii. A low salience or neutral stance towards a frame accounting for the global nature of mineral value chains (represented by low importance or neutral ranking of statements in the categories of responsible sourcing or value chain), production and consumption systems and associated environmental impacts.
iv. A non-existent frame on a holistic approach towards resource governance beyond national interests, silo-thinking and limited institutional capacity called for by academic and civil society stakeholders in contemporary mineral resource governance.

The results show that the most dominant frame is number one. This frame, which aligns with the neoliberal thought, finds particularly many representatives among stakeholder groups of the mining industry, lobby groups, economic chambers, and academics embedded in mining technology research, who traditionally have a strong voice in policy making. Our results point in a somewhat similar direction to previous studies showing that mineral resource policy is considered a means for trade- and industry policy to secure continuing access to mineral deposits and commodity markets (Küblböck 2015, 2020).

Frame three (and to some extent four) is the contrasting frame, emphasising the importance of integrated policies with regards to (i) sectors (secondary and primary raw materials next to environmental considerations); (ii) scales (domestic concerns of resource consumption next to responsible sourcing and value chain concerns on the international level); (iii) targets (mandatory targets for recycling, decreased resource consumption); as well as (iv) stakeholders (broader social stakeholder participation as well as NGO involvement in strategy design). This frame is mainly represented by academia from a non-mining or raw material related background and can be contextualised with discourses of integrated policy-making and sustainable resource governance (Ayuk et al. 2020; Schäfer et al. 2020; Christmann 2021). Aspects of the current scientific discourse around contemporary mineral resource governance are almost entirely absent in the predominant frames, one and two, which reveal only modest aspirations towards a broader and more holistic approach to mineral resource governance:

i. Beyond a narrow view of mineral extraction and secure supply: Frames one and two perceive that mineral extraction and economic growth should be given priority over other means (e.g. environmental protection) without considering a broader view of sustainable and societal development. In contrast, recent academic discourses consistently represent the view that mineral extraction should take a more holistic and integrated approach, i.e. focusing on intergenerational benefits for society and environment even after mining activities ceased (see Ayuk et al. 2020) to earn a SDLO. Similarly, while aspects of mineral resource utilisation (Giurco et al. 2014) and sustainability transitions including the energy transition are not addressed in the dominant frames one and two, they play an increasingly important role in the EU-political debate (Schäfer et al. 2020). This view is in sharp contrast to more critical academic discourses questioning the intent and effect of the energy transition in the context of the current economic paradigm (Lorek and Spangenberg 2014) to pursue a European sustainable development path.
ii. Integrated mineral resource governance: In contrast to the majority of identified frames, integrated approaches towards mineral resource policy are highly relevant in the academic discourse. Several authors highlight the integration of different policy resorts relevant for mineral supply such as environment (Haikola and Anshelm 2016) or land-use planning (Endl et al. 2020). Others point towards generic governance principles such as inclusive design and stakeholder involvement or adaptive capacity (Endl 2017; Hidayat and Pramadi 2019).
iii. Accounting for the global nature of value chains and associated environmental and social considerations: Due to the complexity of international mineral value chains as well as the transboundary environmental and social repercussions of EU resource consumption, contemporary resource governance needs to take global scales into account (Ayuk et al. 2020; Christmann 2021). However, the identified frames relegate topics addressing distributional conflicts regarding benefits and burdens arising from resource use in different parts of the world as lesser concerns than domestic environmental and social matters. While some countries take a more economic approach to securing supply (Barteková and Kemp 2016), Christmann (2021), for example, suggests the EU adopt a more holistic approach to build capacity and strengthen institutions in producer countries or support global governance via the UN. This view aligns with recent critiques of EU level initiatives such as ERMA (Friends of the Earth Europe 2021) and support for domestic mineral extraction (Bolger et al. 2021) without considering the reduction of overall resource consumption (United Nations Environment Programme and International Resource Panel 2011). Ultimately absent in the composition of frames are strong conceptions of ecological justice such as the notion that mineral resource activities should stop when producing a net negative impact on humans and other beings (for legal or constitutional frameworks, see Sbert 2020) or prioritisation of actions to mitigate distributional conflicts and green/resource colonialism (Broad and Fischer-Mackey 2017; Hilson and Maconachie 2020).

The absence of the abovementioned frames may be explained by the invitation policy for the consultation process (Küblböck 2020; Bolger et al. 2021; Friends of the Earth Europe 2021). The small sample size in some of the frames and the strong focus on domestic economy-oriented frames indicate a very narrow selection of participants for the consultation process. Reasons for this limitation can only be assumed, but withdrawal from the initial aim of developing an ‘integrated’ mineral resource strategy suggests that party-political reasons, power struggles, and competing policy priorities (Steurer and Clar 2015; Plank et al. 2021) may explain why the responsible ministry handpicked this selection of participants. Meanwhile, the participation of NGOs (e.g. AG Rohstoffe) or other important ministries (e.g. the Federal Ministry of Austria: Climate Action, Environment, Energy; Mobility, Innovation and Technology) was evidentially considered unimportant. The economic focus in Austrian policy design has also been noted in adjacent policy discourses. For instance, researchers with background in Austria’s climate policy integration illustrate that several ministries and social partners in Austria favour economic growth over other policy priorities and, thus, differ regarding policy priorities on EU level (Kettner and Kletzan‐Slamanig 2020; Plank et al. 2021).

The notable lack of consensus on the vast majority of topics as well as the identified frame conflicts underscore the complex and wicked problem (Rittel and Webber 1973; Endl 2017) of mineral resource governance. Our findings show that these types of problems are further aggravated by clashing discourses as well as values and worldviews of stakeholders that are involved in policy making and mineral resource governance (Sauer and Hiete 2020). We mainly found agreement on issues detached from worldviews or conceptual barriers, such as the agreement that recycling is important. Nevertheless, the fundamental differences between frames suggest that this point of agreement has different underpinnings in each: for frames one and two, recycling represents a new commodity on the global market; frame three likely relates recycling to their desire for overall resource use reduction, while frame four might view recycling and secondary raw materials as opportunities for new business models. While frame conflicts pose substantial challenges for policy making, they are by no means detrimental for policy design (e.g. Davies et al. 2016). A strong frame alignment might lead to the exclusion of stakeholders who dissent, question the overall approach, or ask for systemic change (Mouffe 2005; Metzger 2019), thereby cementing path dependencies and limiting opportunities for progress. Given the benefits of diverse viewpoints in the marketplace of ideas, it is hard to avoid the conclusion that a ‘by-invitation’ consultation process which assembles representatives of a few dominant frames was meant for the advocacy of a certain frame.

## Conclusion

Our research demonstrates the presence of different and to some extent conflicting frames in the consultation process for developing a new mineral resource policy in Austria. We characterise the varying frames and identify possible frame conflicts, which are critical in policy design processes dealing with relevant and contested policy topics such as mineral resources. We point out three main conclusions of our research:

(i) The article demonstrated that Q-methodology is a suitable tool to identify and unravel different frames present in a policy design process. Four different frames were identified, described in detail, and contrasted against each other to reveal potential frame conflicts.
(ii) In an academic world increasingly concerned about the distribution of benefits and burdens along global mineral value chains, the emphasis placed by participants on domestic mineral extraction and the relative neglect of issues in different parts of the world suggest that the dominant frames invited to the consultation process encompass only a narrow sectoral perspective (Ayuk et al. 2020; Christmann 2021).
(iii) For effective policy design it is necessary to either pragmatically resolve or reflect on frame rationality (Schön et al. 1996). Austria was celebrated as a best-practice case in the development of the AMRP 2010 due to engagement, deliberative interaction, and negotiation with different policy actors (Endl 2017). While the implementation of the AMRP 2010 remained fragmented (Gugerell et al. 2020) and also critiqued by the Austrian Court of Auditors (Rechnungshof 2017), the attempt to coordinate and negotiate potentially conflicting policy interests at the time, should be appreciated. From this perspective the current process appears notably less ambitious.

Taking the results presented as a starting point, our next step is to investigate the uptake of these frames in the finalised ‘Austrian Mineral Resource Strategy 2030’ and to assess to which degree frame conflicts have been resolved in the agenda setting process.

## Supplementary Information

Below is the link to the electronic supplementary material.

Supplementary file1 (PDF 237 kb)
