# Supplementary material for: Exploring frame conflicts in the development of a new mineral resource policy in Austria using Q-methodology
Source: Ambio. 2022 Sep 17;52(1):210–28. doi: 10.1007/s13280-022-01761-9 (PMC9483345; doi:10.1007/s13280-022-01761-9)
Supplement: Supplementary file 1 — Supplementary file1 (PDF 237 kb) [file 13280_2022_1761_MOESM1_ESM.pdf]

Ambio

Supplementary Information
This supplementary information has not been peer reviewed.

Title: **Exploring frame conflicts in the development of a new mineral resource policy in Austria using Q-Methodology**

S1 Appendix

Table S1: Full statements of Q-set organized by thematic category

Sub-theme	Statement (statement number indicates the sequence in the Q sort)
Digitalisation	The digitalisation and automation of all players in the value chain of mineral raw materials must be pursued with vigour and implemented quickly. (1)
	I believe that the potential for digitalisation and automation is not being used in Austria. (19)
	Digital interfaces and the efficient digital transfer of information between companies and the authorities involved would improve official procedures. (26)
Recycling/Circular Economy	Recycling should only be carried out if primary raw materials are not available at competitive prices. (9)
	Recycling should be preferred to primary raw materials. (16)
	Substances that are particularly difficult to recycle or components that are particularly harmful to the environment should be replaced as quickly as possible by alternative, more environmentally friendly raw materials or technologies (e.g. replacement of cadmium in batteries). (18)
	Exporting waste/residues to non-European countries with lower social and environmental standards must not be a viable alternative. (33)
	In order to implement (stimulate) the circular economy, product features such as reparability, modular exchange, recycling and upcycling of product parts should already be foreseen in the product design (design for recycling). (37)
	The reuse of recycled raw materials must be defined in such a way that no "downcycling" occurs. (25)
	In a circular economy, primary raw materials will always be needed continuously in the future. (45)
Raw material supply from domestic sources	Ecological, social and economic sustainability in the raw materials sector must be ensured in Austria through high quality production standards in the sense of "responsible production". (13)
	Primary and secondary raw materials extracted in Austria are an important basis for Austria as a business location. (39)
	The fullest possible use of all mineral raw material flows (incl. waste rock, overburden, excavated material, etc.) should be strived for and landfilling should be avoided. (48)
Responsible Sourcing	Austria should participate in the development of more binding due diligence criteria for raw material importing companies. (14)
	An environmentally sound and socially just supply of mineral raw materials gives Austria a competitive advantage. (5)
	In order to withstand international competition, primarily economic aspects play a role for a continuous supply of raw materials. (6)
	At the international level, Austria should use the process initiated in 2014 for a UN treaty on business and human rights to advocate effective, legally binding and globally applicable human rights standards for globally operating companies. (30)
	I think that transparency ends with corporate secrets and commercial company interests. (46)
	I believe that raw material producers and suppliers who do not meet environmental and social standards should be sanctioned and punished. (47)
	Raw materials diplomacy Austria needs border-tax-adjustments that compensate for environmental and social costs. (32)
Value chain	No economic exchange or trade of raw materials should take place with regions that, according to the OECD definition, are conflict-affected and high-risk areas. (2)
	Regional processing of raw materials is necessary to ensure value chains. (10)
	The Conflict Minerals Regulation should be extended to other raw materials. (11)
	Technology leadership should be pursued or promoted in those areas in which Austrian industrial companies already have broad know-how (and possibly "hidden champions"). (38)
	The development of new, sustainable business models should be accelerated to ensure efficient use of resources (e.g. leasing of metals). (21)
Sustainable supply	A prerequisite for circular economy and sustainability is an accurate knowledge of quantitative and qualitative primary and secondary raw material flows. (44)
	Mineral raw materials should be treated equally to renewable raw materials in all aspects. (40)
Land-use planning	Land-use planning plays a central role in ensuring a balance between public and private interests. (20)
	Due to Austria's decentralised structure, the federal provinces and municipalities have a strong role. For a successful implementation of the raw materials strategy, early involvement and good communication with all relevant levels is necessary. (29)
	I am convinced that economically significant mineral raw material deposits must remain accessible for possible future extraction. (31)b
	Spatial planning impedes or prevents the extraction of mineral raw materials. (34)
	In order to improve the acceptance of the extractive sector, it is important to put mineral resources on a higher political agenda. (42)
	Ensuring sustainable mineral extraction and processing is a joint task of companies, politics, administration, education, civil society and the population, research and development. (49)
Ecology/Environment	In official procedures, the public interest in mineral extraction must have the same priority as environmental protection. (3)
	I believe that raw material companies should also be obliged to achieve climate-neutral production by 2050. (7)
	A European energy and mobility transition must not take place at the expense of environmental and social problems elsewhere. (23)
	Extractive sites contribute greatly to biodiversity and ecosystem services. (28)
	We need cross-sectoral (energy, climate, transport, industry, circular economy) policy instruments to reduce EU resource consumption in absolute terms. (36)

	Environmental impacts of all economic/production sectors should be internalised and monetarily compensated (e.g. rubble tax). (41)
	Without legal regulations and possible sanctions, extractive companies are not doing enough to ensure adequate environmental protection and nature conservation. (51)
Social Licence to Operate	I think that the transparent disclosure of projects can generate more social acceptance for raw materials. (8)
	Active participation of the population that goes beyond mere information is an important prerequisite for acceptance at local and overall societal level. (12)
	Mining enjoys a high level of social acceptance in Austria. (17)
	Raising awareness among producers and consumers supports a differentiated discussion on the role of mineral extraction and use (e.g. importance for everyday life, costs, impact on environment/nature). (22)
	Societal demands for sustainable, environmentally sound and ethically justifiable raw materials management are increasing. (27)
	Participation processes should be established in the design of raw material policies and strategies that ensure adequate involvement of civil society. (35)
	I believe that the interests and concerns of business and enterprises are more important than those of NGOs and/or citizens' initiatives. (50)
	The local communities of extractive companies should directly participate in the company's profit. (52)
Others	Stabilisation of commodity prices must be sought, for example through regulations that reduce the influence of commodity speculation. (4)
	Economic, ecological and human rights impact assessments should be carried out in negotiations on trade and investment protection agreements. (16)
	I think that resource reduction and the increase of resource efficiency must be implemented as binding and quantified targets in the Austrian Raw Materials Strategy. (24)
	Development policies need to strengthen partner countries' capacities for effective governance and regulation of the extractive sector. (43)
	I believe that the local population should decide on the use (extraction) of mineral resources/raw materials. (53)
	I think that sustainable supply chains should lead to an equitable distribution of benefits and costs/burdens of the use of raw materials. (54)
